# *N*-(4-Ferrocenylphenyl)benzamide

**DOI:** 10.1107/S1600536810023470

**Published:** 2010-06-23

**Authors:** Ataf Ali Altaf, Amin Badshah, Nasir Khan, M. Nawaz Tahir

**Affiliations:** a Department of Chemistry, Quaid-i-Azam University, Islamabad, Pakistan; b University of Sargodha, Department of Physics, Sargodha, Pakistan

## Abstract

In the title compound, [Fe(C_5_H_5_)(C_18_H_14_NO)], the unsubstituted cyclo­penta­dienyl ring is disordered over two sets of sites with occupancy ratio of 0.55 (1):0.45 (1). One conformation has the rings eclipsed and the other staggered. An intra­molecular C—H⋯O hydrogen bond forms an *S*(6) ring motif. In the crystal, inter­molecular C—H⋯O and N—H⋯O hydrogen bonds lead to *R*_2_^1^(7) ring motifs. The mol­ecules are linked into polymeric chains extending along the *b* axis.

## Related literature

For similar structures, see: Fukuzumi *et al.* (2002 ▶); Shah *et al.* (2007 ▶). For graph-set notation, see: Bernstein *et al.* (1995 ▶).
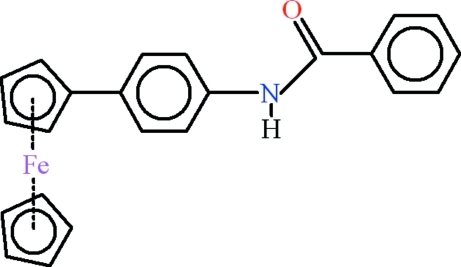


## Experimental

### 

#### Crystal data

[Fe(C_5_H_5_)(C_18_H_14_NO)]
*M* *_r_* = 381.24
Monoclinic, 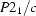

*a* = 20.4467 (16) Å
*b* = 10.3592 (8) Å
*c* = 8.2933 (6) Å
β = 91.996 (3)°
*V* = 1755.6 (2) Å^3^
*Z* = 4
Mo *K*α radiation
μ = 0.87 mm^−1^
*T* = 296 K
0.32 × 0.14 × 0.08 mm

#### Data collection

Bruker Kappa APEXII CCD diffractometer
Absorption correction: multi-scan (*SADABS*; Bruker, 2005 ▶) *T*_min_ = 0.865, *T*_max_ = 0.931
13450 measured reflections
3257 independent reflections
1703 reflections with *I* > 2σ(*I*)
*R*_int_ = 0.074

#### Refinement

*R*[*F* ^2^ > 2σ(*F* ^2^)] = 0.043
*wR*(*F* ^2^) = 0.099
*S* = 1.00
3257 reflections
260 parameters
60 restraints
H-atom parameters constrained
Δρ_max_ = 0.22 e Å^−3^
Δρ_min_ = −0.23 e Å^−3^

### 

Data collection: *APEX2* (Bruker, 2007 ▶); cell refinement: *SAINT* (Bruker, 2007 ▶); data reduction: *SAINT*; program(s) used to solve structure: *SHELXS97* (Sheldrick, 2008 ▶); program(s) used to refine structure: *SHELXL97* (Sheldrick, 2008 ▶); molecular graphics: *ORTEP-3 for Windows* (Farrugia, 1997 ▶) and *PLATON* (Spek, 2009 ▶); software used to prepare material for publication: *WinGX* (Farrugia, 1999 ▶) and *PLATON*.

## Supplementary Material

Crystal structure: contains datablocks global, I. DOI: 10.1107/S1600536810023470/ng2774sup1.cif

Structure factors: contains datablocks I. DOI: 10.1107/S1600536810023470/ng2774Isup2.hkl

Additional supplementary materials:  crystallographic information; 3D view; checkCIF report

## Figures and Tables

**Table:** 

Fe1—C6	2.034 (3)
Fe1—C7	2.030 (4)
Fe1—C8	2.028 (4)
Fe1—C9	2.039 (4)
Fe1—C10	2.059 (3)

**Table:** 

C6—Fe1—C7	40.81 (15)
C6—Fe1—C8	68.11 (17)
C6—Fe1—C9	68.32 (16)
C6—Fe1—C10	40.66 (14)
C7—Fe1—C8	40.32 (18)
C7—Fe1—C9	68.47 (17)
C7—Fe1—C10	68.72 (15)
C8—Fe1—C9	40.79 (16)
C8—Fe1—C10	68.55 (15)
C9—Fe1—C10	40.80 (14)
C1*B*—Fe1—C9	122.2 (3)

**Table 2:** Hydrogen-bond geometry (Å, °)

*D*—H⋯*A*	*D*—H	H⋯*A*	*D*⋯*A*	*D*—H⋯*A*
N1—H1⋯O1^i^	0.86	2.26	3.110 (4)	172
C13—H13⋯O1	0.93	2.48	2.926 (4)	109
C23—H23⋯O1^i^	0.93	2.50	3.180 (4)	130

Symmetry code: (i) 
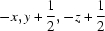
.

**Fig. 1.**
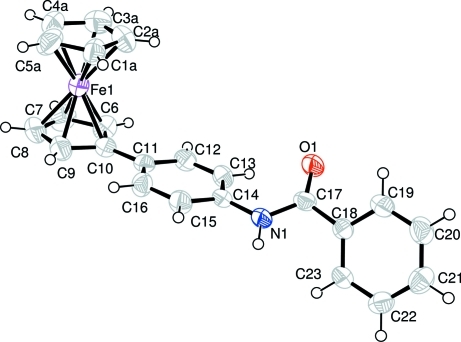
View of (I) with the atom numbering scheme having atoms of greater occupancy ratio. The thermal ellipsoids are drawn at the 50% probability level. H-atoms are shown by small circles of arbitrary radii. The dotted lines indicate the intra-molecular H-bondings.

**Fig. 2.**
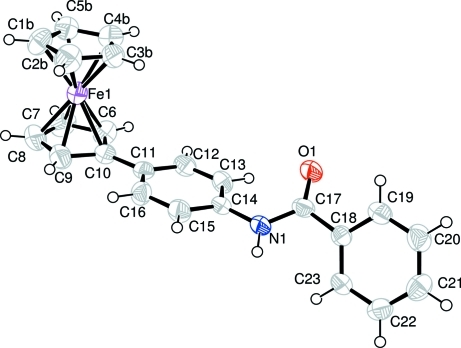
View of (I) with atom numbering scheme having atoms of smaller occupancy ratio.. The thermal ellipsoids are drawn at the 50% probability level. H-atoms are shown by small circles of arbitrary radii. The dotted lines indicate the intra-molecular H-bondings.

**Fig. 3.**
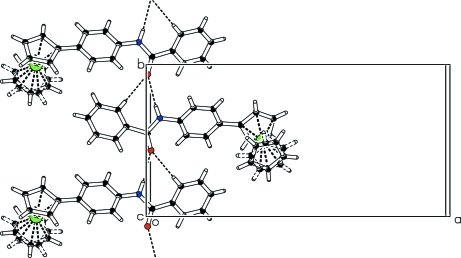
The partial packing (PLATON; Spek, 2009) which shows that molecules form polymeric chains extending along the b axis.

## supplementary crystallographic information

### Comment

The crystal structure of (II) *i.e. N*-(benzylidene)-4-ferrocenylaniline (Shah *et al.*, 2007) and (III) *i.e.* 1-(4-((1,4-Benzoquinonyl)carbonylamino)phenyl)ferrocene (Fukuzumi *et al.*, 2002) have been published. The title compound (I) differs from these due to substituants at the N-atom.

In (I) the cyclopentadienyl ring A (C6–C10), phenyl rings B (C11–C16) and C (C18–C23) are planar with r. m. s. deviations of 0.0024, 0.0020 and 0.0030 Å, respectively. The dihedral angle between A/B is 2.31 (23)° which shows that central phenyl ring is almost planar with attached cyclopentadienyl. The dihedral angle between B/C is 69.05 (8)°. The Fe-atom is at a distance of 1.6435 (17) Å from the centroid of ring A. The important bond distances [Fe–C] and bond angles [C–Fe–C] are given in Table 1. Cyclopentadienyl ring of ferrocene not attached with 4-(benzoylamino)phenyl is disordered over two set of sites with occupancy ratio of 0.548 (14):0.452 (14). There exist intramolecular H-bonding of C—H···O type forming S(6) ring motif (Bernstein *et al.*, 1995). The intermolecular H-bondings of C—H···O and N—H···O type complete *R*_2_^1^(7) ring motif (Table 2, Fig. 2). The molecules are stabilized in the form of polymeric chains extending along the crystallographic *b* axis (Fig. 2). In these chains molecules are connected in helical way due to screw symmetry.

### Experimental

Solution of benzoyl chloride (0.5 ml, 4.296 mmol) in 50 ml anhydrous CHCl_3_ added to the solution of ferrocenyl aniline (1.19 g, 4.296 mmol) and triethylamine (0.71 ml, 5.155 mmol) in 50 ml anhydrous CHCl_3_, at 273 K and stirred for 24 h. The completion of reaction monitored through TLC. To remove extra triethylamine and un-reacted acid chloride and the formed ammonium chloride, the mixture was extracted with distilled water (6 × 100 ml). The solution was evaporated under reduced pressure to give orange solid and re-crystallized form CH_2_Cl_2_. (yield: 84%)

### Refinement

The disordered cyclopentadienyl was refined in two groups as regular pentagons of 1.42 Å. The anisotropic temperature factors of the disordered C atoms were restrained to be nearly isotropic.

The H-atoms were positioned geometrically (N–H = 0.86 Å, C–H = 0.93 Å) and refined as riding with *U*_iso_(H) = x*U*_eq_(C, N), where x = 1.2 for all H-atoms.

### Crystal data

**Table:**

[Fe(C_5_H_5_)(C_18_H_14_NO)]	*F*(000) = 792
*M**_r_* = 381.24	*D*_x_ = 1.442 Mg m^−^^3^
Monoclinic, *P*2_1_/*c*	Mo *K*α radiation, λ = 0.71073 Å
Hall symbol: -P 2ybc	Cell parameters from 1703 reflections
*a* = 20.4467 (16) Å	θ = 2.0–25.5°
*b* = 10.3592 (8) Å	µ = 0.87 mm^−^^1^
*c* = 8.2933 (6) Å	*T* = 296 K
β = 91.996 (3)°	Needle, orange
*V* = 1755.6 (2) Å^3^	0.32 × 0.14 × 0.08 mm
*Z* = 4	

### Data collection

**Table:**

Bruker Kappa APEXII CCD diffractometer	3257 independent reflections
Radiation source: fine-focus sealed tube	1703 reflections with *I* > 2σ(*I*)
graphite	*R*_int_ = 0.074
Detector resolution: 8.20 pixels mm^-1^	θ_max_ = 25.5°, θ_min_ = 2.0°
ω scans	*h* = −24→24
Absorption correction: multi-scan (*SADABS*; Bruker, 2005)	*k* = −11→12
*T*_min_ = 0.865, *T*_max_ = 0.931	*l* = −10→10
13450 measured reflections	

### Refinement

**Table:**

Refinement on *F*^2^	Primary atom site location: structure-invariant direct methods
Least-squares matrix: full	Secondary atom site location: difference Fourier map
*R*[*F*^2^ > 2σ(*F*^2^)] = 0.043	Hydrogen site location: inferred from neighbouring sites
*wR*(*F*^2^) = 0.099	H-atom parameters constrained
*S* = 1.00	*w* = 1/[σ^2^(*F*_o_^2^) + (0.0304*P*)^2^ + 0.2729*P*] where *P* = (*F*_o_^2^ + 2*F*_c_^2^)/3
3257 reflections	(Δ/σ)_max_ < 0.001
260 parameters	Δρ_max_ = 0.22 e Å^−^^3^
60 restraints	Δρ_min_ = −0.23 e Å^−^^3^

### Special details

**Table:**

Geometry. Bond distances, angles *etc*. have been calculated using the rounded fractional coordinates. All su's are estimated from the variances of the (full) variance-covariance matrix. The cell e.s.d.'s are taken into account in the estimation of distances, angles and torsion angles
Refinement. The disordered cyclopentadienyl was refined in two groups as regular pentagons. All the disordered C-atoms were treated anisotropically having equal thermal parameters because refinement anisotropically with individual atoms or rings affoarded large ellipsoids. The sides of regular pentagons after final refinement have naearly 1.392 and 1.436 Å.Refinement of *F*^2^ against ALL reflections. The weighted *R*-factor *wR* and goodness of fit *S* are based on *F*^2^, conventional *R*-factors *R* are based on *F*, with *F* set to zero for negative *F*^2^. The threshold expression of *F*^2^ > σ(*F*^2^) is used only for calculating *R*-factors(gt) *etc*. and is not relevant to the choice of reflections for refinement. *R*-factors based on *F*^2^ are statistically about twice as large as those based on *F*, and *R*- factors based on ALL data will be even larger.

### Fractional atomic coordinates and isotropic or equivalent isotropic displacement parameters (Å^2^)

**Table:**

	*x*	*y*	*z*	*U*_iso_*/*U*_eq_	Occ. (<1)
Fe1	0.37659 (2)	0.49923 (6)	0.17093 (6)	0.0508 (2)	
O1	0.00559 (11)	0.4359 (2)	0.2493 (3)	0.0591 (10)	
N1	0.03504 (13)	0.6469 (3)	0.2387 (3)	0.0431 (10)	
C1A	0.3578 (5)	0.4675 (8)	0.4134 (8)	0.057 (3)	0.548 (14)
C2A	0.3398 (3)	0.3566 (8)	0.3215 (12)	0.062 (4)	0.548 (14)
C3A	0.3962 (6)	0.3122 (6)	0.2429 (9)	0.071 (4)	0.548 (14)
C4A	0.4490 (3)	0.3957 (11)	0.2861 (14)	0.063 (3)	0.548 (14)
C5A	0.4253 (5)	0.4917 (7)	0.3915 (11)	0.071 (3)	0.548 (14)
C6	0.32793 (17)	0.5137 (4)	−0.0465 (4)	0.0573 (14)	
C7	0.3954 (2)	0.5401 (4)	−0.0623 (5)	0.0713 (19)	
C8	0.4114 (2)	0.6458 (4)	0.0363 (6)	0.0715 (19)	
C9	0.35447 (19)	0.6862 (4)	0.1150 (5)	0.0602 (17)	
C10	0.30168 (17)	0.6043 (3)	0.0627 (4)	0.0457 (12)	
C11	0.23316 (16)	0.6128 (3)	0.1118 (4)	0.0391 (12)	
C12	0.18580 (17)	0.5278 (3)	0.0523 (4)	0.0453 (12)	
C13	0.12123 (17)	0.5361 (3)	0.0931 (4)	0.0436 (12)	
C14	0.10100 (16)	0.6315 (3)	0.1977 (4)	0.0380 (11)	
C15	0.14760 (17)	0.7169 (3)	0.2587 (4)	0.0486 (14)	
C16	0.21225 (17)	0.7074 (3)	0.2172 (4)	0.0494 (14)	
C17	−0.00885 (18)	0.5505 (3)	0.2620 (4)	0.0422 (11)	
C18	−0.07618 (16)	0.5899 (3)	0.3012 (4)	0.0370 (11)	
C19	−0.11146 (17)	0.5096 (4)	0.3993 (4)	0.0474 (12)	
C20	−0.17482 (19)	0.5387 (4)	0.4346 (4)	0.0574 (17)	
C21	−0.20407 (18)	0.6490 (4)	0.3725 (4)	0.0555 (16)	
C22	−0.16941 (18)	0.7288 (3)	0.2752 (4)	0.0505 (14)	
C23	−0.10566 (17)	0.7008 (3)	0.2401 (4)	0.0440 (12)	
C4B	0.3690 (6)	0.3127 (5)	0.2407 (9)	0.069 (4)	0.452 (14)
C5B	0.4363 (4)	0.3442 (12)	0.2316 (7)	0.064 (4)	0.452 (14)
C2B	0.3900 (7)	0.4859 (9)	0.4075 (6)	0.071 (4)	0.452 (14)
C3B	0.3403 (3)	0.4003 (14)	0.3494 (7)	0.057 (4)	0.452 (14)
C1B	0.4493 (4)	0.4513 (11)	0.3347 (11)	0.059 (4)	0.452 (14)
H2A	0.29841	0.31948	0.31421	0.0743*	0.548 (14)
H3A	0.39814	0.24098	0.17497	0.0854*	0.548 (14)
H1	0.02125	0.72473	0.24998	0.0516*	
H1A	0.33027	0.51581	0.47674	0.0691*	0.548 (14)
H9	0.35192	0.75418	0.18780	0.0721*	
H12	0.19812	0.46272	−0.01774	0.0542*	
H13	0.09095	0.47731	0.05016	0.0522*	
H15	0.13519	0.78179	0.32884	0.0583*	
H16	0.24256	0.76579	0.26085	0.0595*	
H19	−0.09203	0.43527	0.44165	0.0570*	
H20	−0.19813	0.48397	0.50050	0.0690*	
H21	−0.24698	0.66902	0.39664	0.0667*	
H22	−0.18916	0.80268	0.23229	0.0603*	
H23	−0.08235	0.75637	0.17540	0.0525*	
H4A	0.49163	0.38880	0.25146	0.0754*	0.548 (14)
H5A	0.44968	0.55865	0.43796	0.0843*	0.548 (14)
H6	0.30465	0.44801	−0.09898	0.0682*	
H7	0.42391	0.49520	−0.12674	0.0859*	
H8	0.45265	0.68325	0.04830	0.0857*	
H1B	0.48960	0.49158	0.35164	0.0710*	0.452 (14)
H2B	0.38458	0.55296	0.48043	0.0854*	0.452 (14)
H3B	0.29669	0.40137	0.37753	0.0683*	0.452 (14)
H4B	0.34740	0.24631	0.18514	0.0827*	0.452 (14)
H5B	0.46662	0.30206	0.16913	0.0768*	0.452 (14)

### Atomic displacement parameters (Å^2^)

**Table:**

	*U*^11^	*U*^22^	*U*^33^	*U*^12^	*U*^13^	*U*^23^
Fe1	0.0403 (3)	0.0540 (3)	0.0581 (3)	−0.0033 (3)	0.0001 (2)	0.0041 (3)
O1	0.0488 (16)	0.0309 (15)	0.098 (2)	0.0007 (13)	0.0098 (15)	0.0039 (16)
N1	0.0424 (18)	0.0271 (16)	0.060 (2)	−0.0006 (14)	0.0035 (15)	0.0015 (15)
C1A	0.044 (5)	0.077 (6)	0.051 (5)	−0.007 (5)	0.001 (4)	0.010 (4)
C2A	0.074 (7)	0.052 (6)	0.060 (6)	−0.015 (5)	0.006 (5)	−0.001 (5)
C3A	0.074 (7)	0.058 (6)	0.082 (6)	0.009 (5)	0.001 (5)	0.008 (5)
C4A	0.045 (5)	0.064 (6)	0.080 (7)	0.003 (5)	−0.002 (5)	0.001 (6)
C5A	0.055 (6)	0.088 (6)	0.067 (6)	0.003 (5)	−0.018 (5)	0.004 (6)
C6	0.054 (2)	0.067 (3)	0.051 (2)	0.004 (2)	0.0038 (19)	−0.005 (2)
C7	0.055 (3)	0.091 (4)	0.069 (3)	0.011 (2)	0.020 (2)	0.007 (3)
C8	0.057 (3)	0.064 (3)	0.094 (4)	−0.013 (2)	0.012 (3)	0.018 (3)
C9	0.054 (3)	0.051 (3)	0.076 (3)	−0.006 (2)	0.009 (2)	0.004 (2)
C10	0.047 (2)	0.044 (2)	0.046 (2)	−0.0008 (19)	0.0011 (19)	0.0072 (19)
C11	0.046 (2)	0.035 (2)	0.036 (2)	0.0022 (17)	−0.0020 (18)	0.0062 (17)
C12	0.054 (2)	0.037 (2)	0.045 (2)	−0.0002 (18)	0.0050 (18)	−0.0052 (17)
C13	0.051 (2)	0.038 (2)	0.042 (2)	−0.0072 (16)	0.0026 (18)	−0.0049 (17)
C14	0.041 (2)	0.0288 (19)	0.044 (2)	−0.0018 (16)	0.0004 (18)	0.0049 (18)
C15	0.052 (2)	0.036 (2)	0.058 (3)	−0.0002 (18)	0.004 (2)	−0.0109 (19)
C16	0.041 (2)	0.046 (2)	0.061 (3)	−0.0039 (18)	−0.0027 (19)	−0.011 (2)
C17	0.049 (2)	0.0293 (19)	0.048 (2)	0.0011 (18)	−0.0019 (19)	0.0012 (18)
C18	0.040 (2)	0.0309 (19)	0.040 (2)	−0.0026 (17)	−0.0010 (17)	−0.0052 (17)
C19	0.055 (2)	0.041 (2)	0.046 (2)	−0.003 (2)	0.0010 (17)	0.007 (2)
C20	0.056 (3)	0.064 (3)	0.053 (3)	−0.007 (2)	0.014 (2)	0.009 (2)
C21	0.047 (2)	0.070 (3)	0.050 (3)	0.008 (2)	0.007 (2)	−0.003 (2)
C22	0.049 (2)	0.045 (2)	0.057 (3)	0.008 (2)	−0.004 (2)	−0.002 (2)
C23	0.047 (2)	0.035 (2)	0.050 (2)	−0.0055 (17)	0.0018 (18)	0.0022 (18)
C4B	0.062 (7)	0.065 (7)	0.079 (8)	0.000 (5)	−0.004 (6)	0.015 (6)
C5B	0.059 (7)	0.054 (7)	0.079 (7)	0.001 (5)	−0.007 (6)	0.001 (6)
C2B	0.065 (9)	0.085 (7)	0.064 (6)	0.001 (6)	0.011 (6)	0.001 (6)
C3B	0.050 (6)	0.062 (7)	0.059 (7)	−0.004 (5)	0.006 (5)	0.012 (6)
C1B	0.053 (6)	0.073 (8)	0.051 (7)	0.004 (5)	−0.010 (5)	−0.015 (6)

### Geometric parameters (Å, °)

**Table:**

Fe1—C1A	2.086 (7)	C11—C16	1.390 (5)
Fe1—C2A	2.091 (9)	C12—C13	1.377 (5)
Fe1—C3A	2.063 (7)	C13—C14	1.388 (5)
Fe1—C4A	2.039 (9)	C14—C15	1.383 (5)
Fe1—C5A	2.054 (9)	C15—C16	1.381 (5)
Fe1—C6	2.034 (3)	C17—C18	1.483 (5)
Fe1—C7	2.030 (4)	C18—C23	1.385 (5)
Fe1—C8	2.028 (4)	C18—C19	1.384 (5)
Fe1—C9	2.039 (4)	C19—C20	1.372 (5)
Fe1—C10	2.059 (3)	C20—C21	1.381 (6)
Fe1—C1B	2.040 (9)	C21—C22	1.370 (5)
Fe1—C2B	1.976 (5)	C22—C23	1.376 (5)
Fe1—C3B	1.967 (9)	C1A—H1A	0.9300
Fe1—C4B	2.025 (6)	C1B—H1B	0.9300
Fe1—C5B	2.069 (11)	C2A—H2A	0.9300
O1—C17	1.229 (4)	C2B—H2B	0.9300
N1—C14	1.411 (4)	C3A—H3A	0.9300
N1—C17	1.361 (4)	C3B—H3B	0.9300
N1—H1	0.8600	C4A—H4A	0.9300
C1A—C5A	1.421 (14)	C4B—H4B	0.9300
C1A—C2A	1.420 (12)	C5A—H5A	0.9300
C1B—C2B	1.419 (15)	C5B—H5B	0.9300
C1B—C5B	1.420 (15)	C6—H6	0.9300
C2A—C3A	1.421 (13)	C7—H7	0.9300
C2B—C3B	1.420 (16)	C8—H8	0.9300
C3A—C4A	1.420 (13)	C9—H9	0.9300
C3B—C4B	1.420 (13)	C12—H12	0.9300
C4A—C5A	1.420 (14)	C13—H13	0.9300
C4B—C5B	1.419 (15)	C15—H15	0.9300
C6—C7	1.417 (5)	C16—H16	0.9300
C6—C10	1.422 (5)	C19—H19	0.9300
C7—C8	1.399 (6)	C20—H20	0.9300
C8—C9	1.417 (6)	C21—H21	0.9300
C9—C10	1.428 (5)	C22—H22	0.9300
C10—C11	1.475 (5)	C23—H23	0.9300
C11—C12	1.387 (5)		
			
Fe1···C12	4.001 (4)	C3B···H4B	2.1100
Fe1···C16	4.022 (3)	C3B···H21^iv^	2.9800
Fe1···H1B	2.7100	C4A···H3A	2.1100
Fe1···H2B	2.6300	C4A···H5A	2.1100
Fe1···H3B	2.6100	C4A···H5A^iii^	3.0700
Fe1···H4B	2.6900	C4B···H5B	2.1000
Fe1···H5B	2.7500	C4B···H3B	2.1100
O1···C13	2.926 (4)	C4B···H21^i^	3.0900
O1···C23^i^	3.180 (4)	C5A···H4A	2.1100
O1···N1^i^	3.110 (4)	C5A···H1A	2.1100
O1···H19	2.6000	C5A···H5A^iii^	2.9200
O1···H13	2.4800	C5B···H4B	2.1100
O1···H1^i^	2.2600	C5B···H1B	2.1100
O1···H23^i^	2.5000	C6···H7	2.1000
N1···O1^ii^	3.110 (4)	C6···H12	2.7200
N1···H23	2.6900	C7···H9^v^	3.0800
C1A···C3A	2.298 (11)	C7···H8	2.0800
C1A···C4A	2.298 (12)	C7···H4A^vi^	2.9300
C1A···C9	3.354 (9)	C7···H6	2.1000
C1A···C10	3.399 (8)	C8···H9	2.1000
C1B···C4B	2.297 (14)	C8···H7	2.0900
C1B···C7	3.556 (10)	C9···H8	2.1000
C1B···C8	3.264 (11)	C9···H16	2.7500
C1B···C1B^iii^	3.527 (13)	C9···H2B^v^	3.0000
C1B···C3B	2.298 (11)	C10···H9	2.1100
C1B···C9	3.571 (11)	C10···H16^v^	3.0500
C2A···C4A	2.298 (9)	C10···H6	2.1000
C2A···C6	3.460 (10)	C12···H22^i^	2.9400
C2A···C10	3.418 (9)	C12···H6	2.8900
C2A···C5A	2.298 (12)	C12···H15^v^	2.8700
C2B···C4B	2.297 (10)	C13···H15^v^	2.9100
C2B···C9	3.255 (9)	C14···H19^iv^	3.0800
C2B···C10	3.547 (9)	C15···H20^iv^	3.0400
C2B···C8	3.536 (8)	C15···H19^ii^	3.0100
C2B···C5B	2.297 (13)	C16···H20^iv^	3.0900
C3A···C5A	2.298 (11)	C16···H9	2.9200
C3A···C1A	2.298 (11)	C17···H13^vii^	3.0500
C3A···C7	3.461 (8)	C17···H13	2.8400
C3A···C6	3.440 (9)	C18···H13^vii^	3.0000
C3B···C1B	2.298 (11)	C19···H15^i^	3.0500
C3B···C9	3.560 (13)	C20···H22^viii^	2.9900
C3B···C5B	2.298 (11)	C20···H3B^iv^	3.0500
C3B···C6	3.488 (8)	C21···H22^viii^	3.0300
C3B···C10	3.258 (11)	C21···H3B^iv^	2.9000
C4A···C1A	2.298 (12)	C21···H2A^ii^	3.0000
C4A···C8	3.389 (12)	C22···H2A^ii^	2.8700
C4A···C2A	2.298 (9)	C22···H12^vii^	2.9600
C4A···C7	3.401 (12)	C23···H12^vii^	3.1000
C4B···C7	3.500 (8)	C23···H1	2.6100
C4B···C2B	2.297 (10)	C23···H13^vii^	3.0600
C4B···C1B	2.297 (14)	H1···H15	2.4700
C4B···C6	3.252 (8)	H1···C23	2.6100
C5A···C9	3.344 (10)	H1···H23	2.2100
C5A···C8	3.354 (10)	H1···O1^ii^	2.2600
C5A···C5A^iii^	3.495 (14)	H1B···Fe1	2.7100
C5A···C3A	2.298 (11)	H1B···H1B^iii^	2.4900
C5A···C2A	2.298 (12)	H1B···C1B^iii^	2.9100
C5B···C3B	2.298 (11)	H2A···C22^i^	2.8700
C5B···C2B	2.297 (13)	H2A···C21^i^	3.0000
C5B···C8	3.548 (12)	H2A···H21^i^	2.5400
C5B···C7	3.258 (10)	H2A···H22^i^	2.2600
C5B···C6	3.600 (9)	H2B···Fe1	2.6300
C6···C9	2.287 (6)	H2B···C9^viii^	3.0000
C6···C11	2.590 (5)	H3B···H20^iv^	2.5800
C6···C3A	3.440 (9)	H3B···Fe1	2.6100
C6···C8	2.275 (6)	H3B···H22^i^	2.5600
C6···C2A	3.460 (10)	H3B···C20^iv^	3.0500
C6···C4B	3.252 (8)	H3B···C21^iv^	2.9000
C6···C5B	3.600 (9)	H3B···H21^iv^	2.2800
C6···C3B	3.488 (8)	H4A···H7^vi^	2.3700
C7···C5B	3.258 (10)	H4A···C7^vi^	2.9300
C7···C4A	3.401 (12)	H4B···Fe1	2.6900
C7···C1B	3.556 (10)	H4B···H21^i^	2.2800
C7···C9	2.289 (6)	H5A···C5A^iii^	2.9200
C7···C3A	3.461 (8)	H5A···H5A^iii^	2.5700
C7···C10	2.308 (5)	H5A···C4A^iii^	3.0700
C7···C4B	3.500 (8)	H5B···Fe1	2.7500
C8···C6	2.275 (6)	H5B···H8^vi^	2.4900
C8···C5B	3.548 (12)	H6···C12	2.8900
C8···C1B	3.264 (11)	H6···H12	2.3100
C8···C10	2.302 (5)	H7···H4A^vi^	2.3700
C8···C4A	3.389 (12)	H8···H5B^vi^	2.4900
C8···C2B	3.536 (8)	H9···C7^viii^	3.0800
C8···C5A	3.354 (10)	H9···C16	2.9200
C9···C5A	3.344 (10)	H9···H16	2.3400
C9···C1B	3.571 (11)	H12···C23^vii^	3.1000
C9···C11	2.594 (5)	H12···C6	2.7200
C9···C1A	3.354 (9)	H12···H6	2.3100
C9···C6	2.287 (6)	H12···C22^vii^	2.9600
C9···C3B	3.560 (13)	H13···C23^vii^	3.0600
C9···C2B	3.255 (9)	H13···C17	2.8400
C9···C7	2.289 (6)	H13···C17^vii^	3.0500
C10···C7	2.308 (5)	H13···O1	2.4800
C10···C3B	3.258 (11)	H13···C18^vii^	3.0000
C10···C8	2.302 (5)	H15···C19^ii^	3.0500
C10···C2B	3.547 (9)	H15···C12^viii^	2.8700
C10···C2A	3.418 (9)	H15···H1	2.4700
C10···C1A	3.399 (8)	H15···C13^viii^	2.9100
C12···Fe1	4.001 (4)	H16···C9	2.7500
C12···C22^i^	3.433 (4)	H16···H9	2.3400
C13···C22^i^	3.496 (4)	H16···C10^viii^	3.0500
C13···O1	2.926 (4)	H19···C15^i^	3.0100
C15···C19^ii^	3.375 (5)	H19···C14^iv^	3.0800
C16···Fe1	4.022 (3)	H19···O1	2.6000
C19···C15^i^	3.375 (5)	H20···C15^iv^	3.0400
C22···C12^ii^	3.433 (4)	H20···C16^iv^	3.0900
C22···C13^ii^	3.496 (4)	H20···H3B^iv^	2.5800
C23···O1^ii^	3.180 (4)	H21···C3B^iv^	2.9800
C1A···H2A	2.1100	H21···H3B^iv^	2.2800
C1A···H5A	2.1100	H21···C2A^iv^	3.0700
C1B···H5B	2.1100	H21···H2A^ii^	2.5400
C1B···H2B	2.1100	H21···C4B^ii^	3.0900
C1B···H1B^iii^	2.9100	H21···H4B^ii^	2.2800
C2A···H21^iv^	3.0700	H22···C12^ii^	2.9400
C2A···H1A	2.1100	H22···H2A^ii^	2.2600
C2A···H3A	2.1100	H22···H3B^ii^	2.5600
C2B···H1B	2.1100	H22···C20^v^	2.9900
C2B···H3B	2.1100	H22···C21^v^	3.0300
C3A···H2A	2.1100	H23···N1	2.6900
C3A···H4A	2.1100	H23···H1	2.2100
C3B···H2B	2.1100	H23···O1^ii^	2.5000
			
C1A—Fe1—C2A	39.7 (3)	Fe1—C4B—C5B	71.4 (6)
C1A—Fe1—C3A	67.3 (3)	Fe1—C5A—C4A	69.1 (5)
C1A—Fe1—C4A	67.7 (4)	C1A—C5A—C4A	108.0 (7)
C1A—Fe1—C5A	40.1 (4)	Fe1—C5A—C1A	71.2 (5)
C1A—Fe1—C6	139.9 (3)	Fe1—C5B—C1B	68.7 (6)
C1A—Fe1—C7	177.0 (3)	Fe1—C5B—C4B	68.1 (5)
C1A—Fe1—C8	136.8 (3)	C1B—C5B—C4B	108.0 (7)
C1A—Fe1—C9	108.8 (3)	Fe1—C6—C7	69.4 (2)
C1A—Fe1—C10	110.1 (3)	C7—C6—C10	108.8 (3)
C2A—Fe1—C3A	40.0 (4)	Fe1—C6—C10	70.61 (19)
C2A—Fe1—C4A	67.6 (3)	Fe1—C7—C8	69.8 (3)
C2A—Fe1—C5A	67.3 (3)	Fe1—C7—C6	69.8 (2)
C2A—Fe1—C6	114.0 (3)	C6—C7—C8	107.8 (4)
C2A—Fe1—C7	143.2 (3)	Fe1—C8—C9	70.0 (2)
C2A—Fe1—C8	176.4 (3)	Fe1—C8—C7	69.9 (2)
C2A—Fe1—C9	136.5 (2)	C7—C8—C9	108.7 (4)
C2A—Fe1—C10	110.9 (2)	Fe1—C9—C10	70.4 (2)
C3A—Fe1—C4A	40.5 (4)	C8—C9—C10	108.0 (3)
C3A—Fe1—C5A	67.9 (3)	Fe1—C9—C8	69.2 (2)
C3A—Fe1—C6	114.2 (3)	Fe1—C10—C6	68.73 (19)
C3A—Fe1—C7	115.5 (3)	Fe1—C10—C11	127.6 (2)
C3A—Fe1—C8	142.6 (3)	C6—C10—C9	106.7 (3)
C3A—Fe1—C9	176.0 (3)	Fe1—C10—C9	68.8 (2)
C3A—Fe1—C10	139.2 (3)	C9—C10—C11	126.6 (3)
C4A—Fe1—C5A	40.6 (4)	C6—C10—C11	126.7 (3)
C4A—Fe1—C6	141.2 (3)	C10—C11—C12	121.5 (3)
C4A—Fe1—C7	113.4 (3)	C10—C11—C16	122.1 (3)
C4A—Fe1—C8	112.9 (3)	C12—C11—C16	116.4 (3)
C4A—Fe1—C9	139.3 (3)	C11—C12—C13	122.5 (3)
C4A—Fe1—C10	177.8 (3)	C12—C13—C14	120.5 (3)
C5A—Fe1—C6	177.9 (2)	C13—C14—C15	117.9 (3)
C5A—Fe1—C7	139.0 (3)	N1—C14—C15	119.4 (3)
C5A—Fe1—C8	110.5 (3)	N1—C14—C13	122.6 (3)
C5A—Fe1—C9	109.6 (2)	C14—C15—C16	121.1 (3)
C5A—Fe1—C10	137.6 (3)	C11—C16—C15	121.7 (3)
C6—Fe1—C7	40.81 (15)	O1—C17—N1	122.4 (3)
C6—Fe1—C8	68.11 (17)	O1—C17—C18	120.8 (3)
C6—Fe1—C9	68.32 (16)	N1—C17—C18	116.8 (3)
C6—Fe1—C10	40.66 (14)	C17—C18—C19	118.1 (3)
C1B—Fe1—C6	158.5 (3)	C19—C18—C23	119.0 (3)
C2B—Fe1—C6	158.7 (4)	C17—C18—C23	122.9 (3)
C3B—Fe1—C6	121.3 (2)	C18—C19—C20	120.6 (4)
C4B—Fe1—C6	106.5 (3)	C19—C20—C21	120.2 (4)
C5B—Fe1—C6	122.6 (2)	C20—C21—C22	119.5 (3)
C7—Fe1—C8	40.32 (18)	C21—C22—C23	120.7 (3)
C7—Fe1—C9	68.47 (17)	C18—C23—C22	120.1 (3)
C7—Fe1—C10	68.72 (15)	Fe1—C1A—H1A	127.00
C1B—Fe1—C7	121.8 (3)	C2A—C1A—H1A	126.00
C2B—Fe1—C7	159.4 (4)	C5A—C1A—H1A	126.00
C3B—Fe1—C7	156.2 (3)	C5B—C1B—H1B	126.00
C4B—Fe1—C7	119.4 (2)	Fe1—C1B—H1B	128.00
C5B—Fe1—C7	105.3 (2)	C2B—C1B—H1B	126.00
C8—Fe1—C9	40.79 (16)	Fe1—C2A—H2A	127.00
C8—Fe1—C10	68.55 (15)	C1A—C2A—H2A	126.00
C1B—Fe1—C8	106.7 (3)	C3A—C2A—H2A	126.00
C2B—Fe1—C8	124.0 (3)	C3B—C2B—H2B	126.00
C3B—Fe1—C8	162.4 (4)	Fe1—C2B—H2B	125.00
C4B—Fe1—C8	154.6 (3)	C1B—C2B—H2B	126.00
C5B—Fe1—C8	120.0 (3)	Fe1—C3A—H3A	126.00
C9—Fe1—C10	40.80 (14)	C2A—C3A—H3A	126.00
C1B—Fe1—C9	122.2 (3)	C4A—C3A—H3A	126.00
C2B—Fe1—C9	108.3 (3)	Fe1—C3B—H3B	125.00
C3B—Fe1—C9	125.4 (4)	C4B—C3B—H3B	126.00
C4B—Fe1—C9	162.4 (4)	C2B—C3B—H3B	126.00
C5B—Fe1—C9	156.5 (3)	C3A—C4A—H4A	126.00
C1B—Fe1—C10	158.9 (3)	Fe1—C4A—H4A	125.00
C2B—Fe1—C10	123.0 (4)	C5A—C4A—H4A	126.00
C3B—Fe1—C10	108.0 (3)	Fe1—C4B—H4B	127.00
C4B—Fe1—C10	124.5 (3)	C5B—C4B—H4B	126.00
C5B—Fe1—C10	160.1 (3)	C3B—C4B—H4B	126.00
C1B—Fe1—C2B	41.4 (5)	C1A—C5A—H5A	126.00
C1B—Fe1—C3B	70.0 (3)	Fe1—C5A—H5A	125.00
C1B—Fe1—C4B	68.8 (4)	C4A—C5A—H5A	126.00
C1B—Fe1—C5B	40.4 (4)	Fe1—C5B—H5B	129.00
C2B—Fe1—C3B	42.2 (4)	C4B—C5B—H5B	126.00
C2B—Fe1—C4B	70.1 (3)	C1B—C5B—H5B	126.00
C2B—Fe1—C5B	69.2 (4)	C7—C6—H6	126.00
C3B—Fe1—C4B	41.7 (4)	Fe1—C6—H6	126.00
C3B—Fe1—C5B	69.4 (4)	C10—C6—H6	126.00
C4B—Fe1—C5B	40.5 (4)	C8—C7—H7	126.00
C14—N1—C17	126.3 (3)	Fe1—C7—H7	126.00
C17—N1—H1	117.00	C6—C7—H7	126.00
C14—N1—H1	117.00	C9—C8—H8	126.00
Fe1—C1A—C5A	68.7 (5)	Fe1—C8—H8	126.00
C2A—C1A—C5A	108.0 (7)	C7—C8—H8	126.00
Fe1—C1A—C2A	70.3 (5)	C10—C9—H9	126.00
Fe1—C1B—C2B	66.9 (5)	Fe1—C9—H9	126.00
C2B—C1B—C5B	108.0 (8)	C8—C9—H9	126.00
Fe1—C1B—C5B	70.9 (5)	C13—C12—H12	119.00
Fe1—C2A—C3A	68.9 (4)	C11—C12—H12	119.00
C1A—C2A—C3A	108.0 (7)	C12—C13—H13	120.00
Fe1—C2A—C1A	69.9 (4)	C14—C13—H13	120.00
Fe1—C2B—C1B	71.7 (4)	C14—C15—H15	119.00
Fe1—C2B—C3B	68.5 (4)	C16—C15—H15	119.00
C1B—C2B—C3B	108.1 (7)	C11—C16—H16	119.00
Fe1—C3A—C4A	68.9 (5)	C15—C16—H16	119.00
Fe1—C3A—C2A	71.1 (5)	C18—C19—H19	120.00
C2A—C3A—C4A	108.0 (7)	C20—C19—H19	120.00
Fe1—C3B—C2B	69.3 (5)	C19—C20—H20	120.00
Fe1—C3B—C4B	71.4 (4)	C21—C20—H20	120.00
C2B—C3B—C4B	107.9 (7)	C20—C21—H21	120.00
C3A—C4A—C5A	108.0 (7)	C22—C21—H21	120.00
Fe1—C4A—C5A	70.3 (5)	C21—C22—H22	120.00
Fe1—C4A—C3A	70.7 (5)	C23—C22—H22	120.00
C3B—C4B—C5B	108.0 (7)	C18—C23—H23	120.00
Fe1—C4B—C3B	67.0 (5)	C22—C23—H23	120.00
			
C2A—Fe1—C1A—C5A	119.5 (7)	C6—Fe1—C8—C9	81.7 (3)
C3A—Fe1—C1A—C2A	−37.3 (5)	C7—Fe1—C8—C9	119.8 (4)
C3A—Fe1—C1A—C5A	82.1 (6)	C9—Fe1—C8—C7	−119.8 (4)
C4A—Fe1—C1A—C2A	−81.3 (5)	C10—Fe1—C8—C7	−82.0 (2)
C4A—Fe1—C1A—C5A	38.1 (5)	C10—Fe1—C8—C9	37.8 (2)
C5A—Fe1—C1A—C2A	−119.5 (7)	C1A—Fe1—C9—C8	141.6 (4)
C6—Fe1—C1A—C2A	63.8 (6)	C1A—Fe1—C9—C10	−99.3 (3)
C6—Fe1—C1A—C5A	−176.8 (4)	C2A—Fe1—C9—C8	176.3 (4)
C8—Fe1—C1A—C2A	178.5 (3)	C2A—Fe1—C9—C10	−64.6 (4)
C8—Fe1—C1A—C5A	−62.1 (6)	C4A—Fe1—C9—C8	64.2 (5)
C9—Fe1—C1A—C2A	142.1 (4)	C4A—Fe1—C9—C10	−176.7 (5)
C9—Fe1—C1A—C5A	−98.4 (4)	C5A—Fe1—C9—C8	99.0 (4)
C10—Fe1—C1A—C2A	98.8 (4)	C5A—Fe1—C9—C10	−141.9 (3)
C10—Fe1—C1A—C5A	−141.8 (4)	C6—Fe1—C9—C8	−81.2 (3)
C1A—Fe1—C2A—C3A	−119.6 (7)	C6—Fe1—C9—C10	37.9 (2)
C3A—Fe1—C2A—C1A	119.6 (7)	C7—Fe1—C9—C8	−37.1 (3)
C4A—Fe1—C2A—C1A	81.6 (6)	C7—Fe1—C9—C10	82.0 (2)
C4A—Fe1—C2A—C3A	−38.0 (6)	C8—Fe1—C9—C10	119.1 (3)
C5A—Fe1—C2A—C1A	37.5 (5)	C10—Fe1—C9—C8	−119.1 (3)
C5A—Fe1—C2A—C3A	−82.1 (5)	C1A—Fe1—C10—C6	−145.5 (3)
C6—Fe1—C2A—C1A	−140.8 (5)	C1A—Fe1—C10—C9	95.8 (3)
C6—Fe1—C2A—C3A	99.7 (5)	C1A—Fe1—C10—C11	−24.8 (4)
C7—Fe1—C2A—C1A	−178.8 (5)	C2A—Fe1—C10—C6	−102.9 (3)
C7—Fe1—C2A—C3A	61.7 (6)	C2A—Fe1—C10—C9	138.3 (3)
C9—Fe1—C2A—C1A	−57.6 (6)	C2A—Fe1—C10—C11	17.8 (4)
C9—Fe1—C2A—C3A	−177.2 (4)	C3A—Fe1—C10—C6	−67.3 (4)
C10—Fe1—C2A—C1A	−96.8 (5)	C3A—Fe1—C10—C9	173.9 (4)
C10—Fe1—C2A—C3A	143.7 (5)	C3A—Fe1—C10—C11	53.4 (5)
C1A—Fe1—C3A—C2A	37.1 (5)	C5A—Fe1—C10—C6	178.3 (4)
C1A—Fe1—C3A—C4A	−81.8 (6)	C5A—Fe1—C10—C9	59.5 (4)
C2A—Fe1—C3A—C4A	−118.9 (7)	C5A—Fe1—C10—C11	−61.0 (5)
C4A—Fe1—C3A—C2A	118.9 (7)	C6—Fe1—C10—C9	−118.8 (3)
C5A—Fe1—C3A—C2A	80.7 (5)	C6—Fe1—C10—C11	120.7 (4)
C5A—Fe1—C3A—C4A	−38.2 (6)	C7—Fe1—C10—C6	37.5 (2)
C6—Fe1—C3A—C2A	−99.1 (5)	C7—Fe1—C10—C9	−81.3 (2)
C6—Fe1—C3A—C4A	142.1 (5)	C7—Fe1—C10—C11	158.2 (3)
C7—Fe1—C3A—C2A	−144.2 (4)	C8—Fe1—C10—C6	80.9 (2)
C7—Fe1—C3A—C4A	96.9 (5)	C8—Fe1—C10—C9	−37.8 (2)
C8—Fe1—C3A—C2A	175.9 (4)	C8—Fe1—C10—C11	−158.4 (3)
C8—Fe1—C3A—C4A	57.0 (7)	C9—Fe1—C10—C6	118.8 (3)
C10—Fe1—C3A—C2A	−57.8 (6)	C9—Fe1—C10—C11	−120.5 (4)
C10—Fe1—C3A—C4A	−176.7 (5)	C17—N1—C14—C13	37.2 (5)
C1A—Fe1—C4A—C3A	80.6 (6)	C17—N1—C14—C15	−145.1 (3)
C1A—Fe1—C4A—C5A	−37.7 (5)	C14—N1—C17—O1	0.2 (5)
C2A—Fe1—C4A—C3A	37.5 (5)	C14—N1—C17—C18	−178.8 (3)
C2A—Fe1—C4A—C5A	−80.8 (6)	Fe1—C1A—C2A—C3A	58.6 (6)
C3A—Fe1—C4A—C5A	−118.3 (8)	C5A—C1A—C2A—Fe1	−58.5 (5)
C5A—Fe1—C4A—C3A	118.3 (8)	C5A—C1A—C2A—C3A	0.0 (9)
C6—Fe1—C4A—C3A	−63.4 (7)	Fe1—C1A—C5A—C4A	−59.6 (7)
C6—Fe1—C4A—C5A	178.3 (4)	C2A—C1A—C5A—Fe1	59.6 (6)
C7—Fe1—C4A—C3A	−102.4 (5)	C2A—C1A—C5A—C4A	0.0 (10)
C7—Fe1—C4A—C5A	139.3 (5)	Fe1—C2A—C3A—C4A	59.2 (6)
C8—Fe1—C4A—C3A	−146.4 (5)	C1A—C2A—C3A—Fe1	−59.2 (6)
C8—Fe1—C4A—C5A	95.3 (5)	C1A—C2A—C3A—C4A	−0.1 (10)
C9—Fe1—C4A—C3A	173.9 (4)	Fe1—C3A—C4A—C5A	60.6 (7)
C9—Fe1—C4A—C5A	55.6 (7)	C2A—C3A—C4A—Fe1	−60.6 (6)
C1A—Fe1—C5A—C4A	118.6 (7)	C2A—C3A—C4A—C5A	0.1 (11)
C2A—Fe1—C5A—C1A	−37.1 (5)	Fe1—C4A—C5A—C1A	60.9 (6)
C2A—Fe1—C5A—C4A	81.5 (5)	C3A—C4A—C5A—Fe1	−60.9 (7)
C3A—Fe1—C5A—C1A	−80.5 (6)	C3A—C4A—C5A—C1A	0.0 (11)
C3A—Fe1—C5A—C4A	38.1 (6)	Fe1—C6—C7—C8	−59.7 (3)
C4A—Fe1—C5A—C1A	−118.6 (7)	C10—C6—C7—Fe1	59.9 (2)
C7—Fe1—C5A—C1A	175.6 (4)	C10—C6—C7—C8	0.2 (5)
C7—Fe1—C5A—C4A	−65.8 (6)	Fe1—C6—C10—C9	58.6 (3)
C8—Fe1—C5A—C1A	139.7 (4)	Fe1—C6—C10—C11	−121.8 (3)
C8—Fe1—C5A—C4A	−101.6 (5)	C7—C6—C10—Fe1	−59.1 (3)
C9—Fe1—C5A—C1A	96.2 (4)	C7—C6—C10—C9	−0.6 (4)
C9—Fe1—C5A—C4A	−145.2 (5)	C7—C6—C10—C11	179.1 (3)
C10—Fe1—C5A—C1A	59.5 (6)	Fe1—C7—C8—C9	−59.4 (3)
C10—Fe1—C5A—C4A	178.1 (4)	C6—C7—C8—Fe1	59.7 (3)
C1A—Fe1—C6—C7	175.5 (4)	C6—C7—C8—C9	0.2 (5)
C1A—Fe1—C6—C10	55.7 (5)	Fe1—C8—C9—C10	−59.9 (3)
C2A—Fe1—C6—C7	−145.6 (3)	C7—C8—C9—Fe1	59.4 (3)
C2A—Fe1—C6—C10	94.6 (3)	C7—C8—C9—C10	−0.6 (5)
C3A—Fe1—C6—C7	−101.6 (4)	Fe1—C9—C10—C6	−58.5 (2)
C3A—Fe1—C6—C10	138.6 (4)	Fe1—C9—C10—C11	121.9 (3)
C4A—Fe1—C6—C7	−62.1 (5)	C8—C9—C10—Fe1	59.2 (3)
C4A—Fe1—C6—C10	178.2 (4)	C8—C9—C10—C6	0.7 (4)
C7—Fe1—C6—C10	−119.8 (3)	C8—C9—C10—C11	−179.0 (3)
C8—Fe1—C6—C7	37.6 (2)	Fe1—C10—C11—C12	−90.0 (4)
C8—Fe1—C6—C10	−82.1 (2)	Fe1—C10—C11—C16	91.2 (4)
C9—Fe1—C6—C7	81.7 (3)	C6—C10—C11—C12	0.2 (5)
C9—Fe1—C6—C10	−38.0 (2)	C6—C10—C11—C16	−178.5 (3)
C10—Fe1—C6—C7	119.8 (3)	C9—C10—C11—C12	179.7 (3)
C2A—Fe1—C7—C6	59.4 (4)	C9—C10—C11—C16	1.0 (5)
C2A—Fe1—C7—C8	178.3 (4)	C10—C11—C12—C13	−178.2 (3)
C3A—Fe1—C7—C6	98.2 (4)	C16—C11—C12—C13	0.6 (5)
C3A—Fe1—C7—C8	−142.9 (4)	C10—C11—C16—C15	178.1 (3)
C4A—Fe1—C7—C6	142.8 (4)	C12—C11—C16—C15	−0.7 (5)
C4A—Fe1—C7—C8	−98.3 (4)	C11—C12—C13—C14	−0.2 (5)
C5A—Fe1—C7—C6	−176.9 (4)	C12—C13—C14—N1	177.7 (3)
C5A—Fe1—C7—C8	−58.0 (4)	C12—C13—C14—C15	0.0 (5)
C6—Fe1—C7—C8	118.9 (3)	N1—C14—C15—C16	−177.9 (3)
C8—Fe1—C7—C6	−118.9 (3)	C13—C14—C15—C16	−0.2 (5)
C9—Fe1—C7—C6	−81.3 (3)	C14—C15—C16—C11	0.6 (5)
C9—Fe1—C7—C8	37.5 (2)	O1—C17—C18—C19	32.4 (5)
C10—Fe1—C7—C6	−37.4 (2)	O1—C17—C18—C23	−145.5 (3)
C10—Fe1—C7—C8	81.5 (2)	N1—C17—C18—C19	−148.6 (3)
C1A—Fe1—C8—C7	−179.0 (4)	N1—C17—C18—C23	33.5 (5)
C1A—Fe1—C8—C9	−59.2 (5)	C17—C18—C19—C20	−177.5 (3)
C3A—Fe1—C8—C7	63.6 (5)	C23—C18—C19—C20	0.5 (5)
C3A—Fe1—C8—C9	−176.6 (4)	C17—C18—C23—C22	177.0 (3)
C4A—Fe1—C8—C7	99.8 (4)	C19—C18—C23—C22	−1.0 (5)
C4A—Fe1—C8—C9	−140.4 (4)	C18—C19—C20—C21	−0.1 (5)
C5A—Fe1—C8—C7	143.6 (3)	C19—C20—C21—C22	0.2 (5)
C5A—Fe1—C8—C9	−96.6 (4)	C20—C21—C22—C23	−0.6 (5)
C6—Fe1—C8—C7	−38.1 (2)	C21—C22—C23—C18	1.0 (5)

Symmetry codes: (i) −*x*, *y*−1/2, −*z*+1/2; (ii) −*x*, *y*+1/2, −*z*+1/2; (iii) −*x*+1, −*y*+1, −*z*+1; (iv) −*x*, −*y*+1, −*z*+1; (v) *x*, −*y*+3/2, *z*−1/2; (vi) −*x*+1, −*y*+1, −*z*; (vii) −*x*, −*y*+1, −*z*; (viii) *x*, −*y*+3/2, *z*+1/2.

### Hydrogen-bond geometry (Å, °)

**Table:**

*D*—H···*A*	*D*—H	H···*A*	*D*···*A*	*D*—H···*A*
N1—H1···O1^ii^	0.8600	2.2600	3.110 (4)	172.00
C13—H13···O1	0.9300	2.4800	2.926 (4)	109.00
C23—H23···O1^ii^	0.9300	2.5000	3.180 (4)	130.00

Symmetry codes: (ii) −*x*, *y*+1/2, −*z*+1/2.
